# PROTOCOL: The experiences of adults experiencing homelessness when accessing and using psychosocial interventions: A systematic review and qualitative evidence synthesis

**DOI:** 10.1002/cl2.1289

**Published:** 2022-11-23

**Authors:** Chris O'Leary, Anton Roberts, Ligia Teixeira, Esther Coren

**Affiliations:** ^1^ Policy Evaluation and Research Unit Manchester Metropolitan University Manchester UK; ^2^ Centre for Homelessness Impact London UK; ^3^ Canterbury Christchurch University Canterbury UK

## Abstract

The systematic review set out in this protocol is part of a broader evidence synthesis which intends to produce two systematic reviews to address a significant gap in the evidence base identified by Luchenski et al. (2018) and by White and Narayanan (2021). This review (the focus of this protocol) will be of the experiences of adults experiencing homelessness when accessing and using psychosocial interventions. This review of qualitative data will use thematic synthesis to analyse these experiences as faced by this population when accessing and using psychosocial interventions.

## 1 BACKGROUND

### 1.1 The problem, condition or issue

#### 1.1.1 The significant and increasing scale of homelessness

Homelessness is a major social and public health concern (MacKnee & Mervyn, 2002; Wright, 2017). In recent years, rates of homelessness are reported to have increased in many western countries, although differences in definitions and measures mean that it is challenging to get an accurate overall picture (OECD, 2020). For example, in the United States, the recent *State of Homelessness in America* report stated that in January 2020 over 580,000 were experiencing homelessness, and that rates of homelessness had grown by 2% over the previous year (National Alliance to End Homelessness, 2021). In Canada, around 35,000 people are homeless each night, with between 250,000 and 300,000 experiencing homelessness a year (Gaetz et al., 2016; Wong et al., 2020). Homelessness continues to rise in most European Union (EU) countries (FEANTSA, 2017). In England, all forms of homelessness rose between 2008 and 2017 (O'Leary & Simcock, 2020), and it is estimated that 280,000 people are homeless in England (Shelter, 2021). Recent published data suggests that the number of people experiencing street homelessness and who are sleeping rough (unsheltered) in England fell between 2017 and 2021 (snapshot count taken on a single night in Autumn), with a significant fall recorded in 2020. The large drop in 2020 is probably accounted for by government responses to the Covid 19 (DLUHC, 2022), though the reasons for reductions in 2017, 2018 and 2019 are not yet known. In the UK, the proportion of people experiencing homelessness who are sleeping rough is relatively small compared to other forms of homelessness. The upward trend has continued in these other types of homelessness, with the number of households assessed as being statutory homeless (i.e., meet the legal definition of homelessness to whom local housing authorities owe a duty to support) has continued to increase (DLUHC, 2022).

We recognise that homelessness is a complex and multifaceted concept, with differences in how homelessness is understood and experienced, and how these differences are conceptualised and described. There are also ongoing policy and practice debates regarding the causes of homelessness, and around interventions aimed at preventing and reducing homelessness. In terms of the causes of homelessness, Bramley and Fitzpatrick state that there is significant debate about whether research on homelessness should focus on individual‐level risks or causes, and structural or systemic causes (such as labour market conditions, housing supply, and poverty). These foci vary between countries and over time, though increasingly it is recognised that both might have explanatory power (Bramley & Fitzpatrick, 2018). These debates often influence policy choices regarding the types of interventions that might address homelessness, and whether these should be focused on structural interventions such as increasing housing supply or reducing poverty or preventing/addressing homelessness at the level of the individual. Whilst individual experiences are highly likely impacted by the structural contexts in which they arise, this review is focused on individual level interventions.

Homelessness is a traumatic experience, which can have a devastating effect on those experiencing it. Several studies, some of which are cited below, have highlighted that more visible and extreme forms of homelessness are often associated with adverse childhood events (Koh & Montgomery, 2021), extreme social disadvantage (Mabhala et al., 2017), physical, emotional and sexual abuse (Green et al., 2012; Henny & Kidler, 2007), neglect (Mar et al., 2019), low self‐esteem (Seale et al., 2016), poor physical and mental health (Vallesi et al., 2021), and much lower life expectancy compared to the general population (ONS, 2019). People experiencing these more extreme and visible forms of homelessness often experience severe and multiple disadvantages (Bramley et al., 2020) and need significant levels of professional and service support (Dobson, 2019). They are increasingly the focus of policy interest, both in the UK and elsewhere, and there is a growing recognition that ‘groups experiencing problems such as homelessness, drug and alcohol misuse, poor mental health, and offending behaviours are often populated to a large extent by the same people’ (Bramley et al., 2020, p. 390). They often face a ‘tri‐morbidity’ (Cornes et al., 2018); a combination of poor physical health, mental health, and problematic substance use (Cornes et al., 2018; Dobson, 2019; Fitzpatrick et al., 2013; Luchenski et al., 2018; Renedo & Jovchelovitch, 2007). It is also the case that longer periods of homelessness may be associated with greater severity of these issues (Mayock et al., 2011).

It is increasingly recognised that this group of adults experiencing homelessness face significant barriers accessing services, and often fall through the cracks between different services they need to access (Dobson, 2019). They have repeated, but intermittent, contact with a range of publicly funded services, particularly health (Aldridge et al., 2018), criminal justice (Bramley et al., 2020), and local government (Dobson, 2019). For example, this population is five times more likely to attend Accident and Emergency (Emergency Room), and three times more likely to be admitted to hospital, than their housed peers (Cornes et al., 2018). It is therefore essential to understand what barriers adults experiencing homelessness face when they access psychosocial interventions, and what might prevent or reduce these interventions from being effective.

It is increasingly recognised that people experiencing homelessness (and particularly those experiencing more extreme and visible forms of homelessness) face significant barriers accessing services, and often fall through the cracks between different services they need to access (Dobson, 2019). They can have repeated, but intermittent, contact with a range of publicly funded services, particularly health (Aldridge et al., 2018), criminal justice (Bramley et al., 2020), and local government (Dobson, 2019).

Psychosocial interventions are aimed at reducing or preventing harms caused by homelessness, and addressing issues that increase the risk of an individual becoming homeless. These interventions are increasingly used with this population for several reasons. First, there is growing evidence of their effectiveness more generally, and it is assumed they must therefore be effective for people experiencing homelessness. However, this assumption may not be valid, given that people experiencing homelessness face significant challenges when accessing, maintaining, and benefiting from services compared to the general population. All things being equal, it is reasonable to assume that given these challenges, evidence about the effectiveness in general of these interventions is not directly translatable to this specific population of people experiencing homelessness. The purpose of the review proposed here is to systematically identify and synthesise evidence of these challenges, so that policy makers and practitioners can take them into account when designing and delivering psychosocial interventions for adults experiencing homelessness. Secondly, because these types of intervention are often used to address clinical needs (such as mental health and substance use) with which people experiencing homelessness often present. Finally, a number of health bodies (e.g., NICE in the UK) recommend their use.

### 1.2 The intervention

#### 1.2.1 Defining psychosocial interventions

There is a lack of a single, agreed definition of psychosocial interventions (Hodges et al., 2011). In a recent Cochrane systematic review of psychosocial interventions for informal (i.e., unpaid family or friends) caregivers, Treanor et al. (2019) set out their own definition as ‘focused on non‐pharmacological interventions that were designed to inform, educate and increase the coping capacity’ of the intervention's recipient. In another systematic review about the effectiveness of psychosocial interventions for depression in older people, Forsman et al. (2011) drew on a definition of an earlier systematic review (Ruddy & House, 2005) that ‘any intervention that emphasizes psychological or social factors rather than biological factors’, which they state includes ‘psychological interventions and health education, as well as interventions with a focus on social aspects, such as social support’. Another definition by Jhanjee (2014) states that psychosocial interventions are ‘…a broad array of treatment interventions, which have varied theoretical backgrounds. They are aimed at eliciting changes in the patient's drug use behaviors well as other factors such as cognition and emotion using the interaction between therapist and patient’ (p. 112). The Welsh Government defined psychosocial interventions as: ‘…therapeutic and structured processes, which address the psychological and social aspects of behaviour. The interventions can vary in intensity depending on the needs of individuals’ (Welsh Government, 2011). One broad definition, and the definition that we propose to use for this review, is provided by England et al. (2015) in their report *Psychosocial interventions for mental and substance use disorders: a framework for establishing evidence‐based standards*. This report was the outcome of detailed work by a committee of 16 experts established by the Institute of Medicine in the United States. In the report, Mary England and colleagues state that psychosocial interventions are ‘interpersonal or informational activities, techniques, or strategies that target biological, behavioral, cognitive, emotional, interpersonal, social, or environmental factors’ (England et al., 2015, p. 5) which aim to make positive changes to the lives of individuals engaging in these activities.

There are some commonalities underpinning these various definitions. These include interventions have a change objective/aim, and that this intended change is psychological, and is often (though not exclusively) focused on mental health or substance use. Several include social change as well as psychological change as an objective, and also all exclude interventions that are wholly or mostly pharmacological in approach. But the extant literature also identifies huge variation in these interventions, including differences in setting, intensity, whether the intervention is group or individual based, and the treatment goals of the intervention. For this review, we propose to use the definition provided by England et al. (2015) and outlined above, and to focus on psychosocial interventions that are: (a) structured or planned, with an explicit intended goal or objective; (b) excludes pharmacological interventions (or interventions that are predominately pharmacological in nature); and (c) targeted for use with adults experiencing homelessness.

#### 1.2.2 Psychosocial interventions and adults experiencing homelessness

Psychosocial interventions are often used to address problematic substance use, poor mental health, and offending behaviours, as well wider social determinants of health such as housing instability and homelessness, worklessness, and poor skills or education. As adults experiencing homelessness may be dealing with more than one of these issues at any given time, many will access services that include psychosocial interventions. It is therefore essential to understand what barriers adults experiencing homelessness might face when accessing these interventions, and what might facilitate improved access. It is also important to understand what factors adults experiencing homelessness perceive in relation to the effectiveness for them, of these interventions.

#### 1.2.3 How the intervention might work

Broadly speaking, the main mechanism of change underpinning these interventions is psychological, focusing on the individual's psychological development and interaction with their social environment. There is no single theory of change underpinning these types of interventions; some are more explicitly based on formal theories, others less so. England et al. (2015) and others argue that psychosocial interventions draw on different theoretical models. In some areas, there are many different interventions derived from the same theoretical model. They also suggest that a number of interventions are adaptations of other interventions targeting different ages, delivery methods (e.g., individual, group), or settings. At this stage, we draw on three broad theories of change to understand how psychosocial interventions might work. These theories of change will be further developed and critically evaluated through the early stages of the evidence synthesis and incorporated where relevant within the analysis framework. The three broad theories of change are:

- Interpersonal relationships. This assumes that an individual's interactions with other people affect their sense of security, self, motivations, physical health, and behaviours. The idea is that an individual's current relationships drive homelessness, substance use, and mental health issues. Psychosocial interventions drawing on this approach focusing on improving interpersonal functioning, providing effective tools for dealing with relationship problems. They also involve providing supportive, non‐judgement support. Family therapy is an example of an intervention that draws on this theory of change.
- Habituation. Habituation assumes that, over time, certain behaviours change from being reward‐driven to be automatized, highly stimulus bound, inflexible, and insensitive to the associated outcomes (positive or negative). Psychosocial interventions drawing on this approach aim to disrupt of change these habits, using approaches such as exposure therapy or contingency rewards.
- Meta cognitive awareness refers to a set of activities which involve thinking about one's thinking and responding accordingly to what is happening in the moment in one's life. Psychosocial interventions drawing on metacognitive awareness approaches focus on cognitive processes and related dysfunctional beliefs or specific cognitive biases. The aim is to help individuals understand how their cognitive biases might lead to, or prolong, homelessness, substance use, and mental health issues, and to provide alternative ways of responding to these thoughts and thereby reduce these symptoms. Motivational interviewing is an example of an intervention that draws on this theory of change.

It is possible that some individual interventions might draw on more than one of these theories of change. As the review team develops and critically assesses these theories of change, it will need to identify which specific interventions draw on which theory, and whether any draw on more than one theory of change.

### 1.3 Why it is important to do this review

#### 1.3.1 Policy relevance

As noted above in the initial introduction, and elsewhere in the background sections, homelessness is a significant and growing policy issue in a number of high income countries around the world. It is increasingly recognised that homelessness has a devastating effect on those experiencing it and on the wider community, and that is costly to the public purse. There is ongoing uncertainty as to which interventions are most effective at preventing and reducing homelessness and the harms associated with homelessness, particularly in relation to people experiencing severe and multiple disadvantage homelessness.

Psychosocial interventions increasingly play a role in policy and practice responses to homelessness and the harms caused by homelessness. There is some evidence about the effectiveness of these interventions generally, but not specifically in relation to adults experiencing homelessness. There is also a limited but growing evidence base factors affecting access and use of psychosocial intervention, but there is no evidence specifically related to adults experiencing homelessness. For example, Troup et al. (2021) recently published a systematic review on the barriers and facilitators faced when scaling up psychosocial interventions during humanitarian crises. This systematic review undertook narrative synthesis of 14 eligible studies. Several studies examine psychosocial interventions to address mental health issues (e.g., Velasco et al., 2020; Raphael et al., 2021, including one in relation to dementia [Rapaport et al., 2017]). Most of these existing studies are not directly relevant to the population of adults experiencing homelessness.

There is also a significant gap in the current evidence base in terms of the voice of people with lived experience of homelessness, as it largely treats people with lived experience as passive research participants. This proposed review aims to elevate the voice of people with lived experience in three ways. First, there will be an ‘experts by experience’ review process that will run alongside the technical peer review process. This will enable the review team to gain views on relevance and appropriateness of the review and its outcomes to the users of services. Secondly, the team proposes to work with a panel of people with lived experience to co‐produce the discussion, recommendations, and conclusions of the published review. Thirdly, this review focuses specifically on the experiences of people experiencing homelessness as they access and use psychosocial interventions and thus aims to hear directly the voice of homeless people as collected in the included studies. We will use the Guidance for Reporting Involvement of Patients and the Public (GRIPP2) (Staniszewska et al., 2017) process to report how we engaged with people experiencing homelessness in the design, conduct, reporting, and developing policy and practice recommendations arising from this review.

#### 1.3.2 Previous reviews

There are no systematic reviews that focus on the experiences of accessing or using, or the effectiveness of psychosocial interventions for this population. One review published by Carver et al. (2020) on interventions aimed at addressing problematic substance use by people experiencing homelessness, which examines study participants’ perceptions of what makes for effective interventions, using meta‐ethnography to synthesise findings. The review proposed here is different in several ways to the review completed by Carver et al. both in terms of its scope and methods. There is some crossover between the two reviews, as many psychosocial interventions are aimed at addressing problematic substance use. However, the proposed review will also consider psychosocial interventions in relation to mental health and housing stability. Our review also only focuses on psychosocial interventions, whereas the Carver review examined a number of other intervention types. As such, the review team believes that this proposed review complements, rather than replicates, the review published by Hannah Carver and colleagues.

## 2 OBJECTIVES

The systematic review set out in this protocol is part of a broader evidence synthesis which intends to produce two systematic reviews to address a significant gap in the evidence base identified by Luchenski et al. (2018) and by White and Narayanan (2021). This review (the focus of this protocol) will be of the experiences of adults experiencing homelessness when accessing and using psychosocial interventions. This review of qualitative data will use thematic synthesis to analyse these experiences as faced by this population when accessing and using psychosocial interventions.

The second review (which is covered by a separate title registration and protocol) will use meta‐analysis to synthesise the effectiveness of different psychosocial interventions in (1) reducing problematic substance use; (2) improving mental health; and (3) improving housing stability for adults experiencing homelessness. Housing is recognised as a significant social determinant of health (Mwoka et al., 2021; Rolfe et al., 2020), but is not addressed as such in the wider literature.

This review will aim to answer the following research questions:

1. What are the experiences of study participants when accessing or using psychosocial interventions?
2. Whether and how adults experiencing homelessness perceive the interventions work for them?
3. To what extent do these experiences vary by type of intervention, context, setting, geographical location, whether individual or group based, whether stand alone or integrated with other interventions?
4. What are the explicit theories of change underpinning psychosocial interventions?

## 3 METHODS

### 3.1 Criteria for considering studies for this review

The SPIDER framework (Cooke et al., 2012) was used in the development of the criteria for considering studies for this review. The following paragraphs set out the **S**ample, **P**henomenon of **I**nterest, **D**esign, **E**valuation, **R**esearch type.

#### 3.1.1 Sample

There are a number of definitions of homelessness available, reflecting differences between countries and over time. There are also different forms of homelessness, taking into account the length of time someone has been experiencing homelessness, distinctions between living on the street or in their vehicles, or having a temporary place to stay.

We propose to draw on the definition of homelessness used by Keenan et al. (2020) in a recently published Campbell Collaboration protocol. During the scoping work to develop this protocol, a workshop of five individuals with lived experienced of homelessness was convened to consider definitions, criteria for inclusion and exclusion, and the process of conducting this review. Following recommendations from those involved in this workshop, we have slightly adapted and widened the definition of homelessness developed by Keenan et al. (2020). The revised definition for this review is:

Homelessness is defined as those individuals who are in inadequate accommodation (environments which are unhygienic and/or overcrowded), who are sleeping rough (sometimes defined as street homeless or unsheltered), those in temporary accommodation (such as shelters and hostels), those in insecure accommodation (such as those facing eviction or in abusive or unsafe environments), and people whose accommodation is inappropriate (such as those living in tents or vehicles, or ‘sofa surfing’).

Our focus is on adults (men and women aged 18 years and over), undertaken in any high‐income country and published in English. Studies of families or children will be excluded from the review. In many countries (particularly the UK), there are different legal frameworks that apply to homeless families and children, and thereby their access to different types of services, and different outcomes expected.

The review will include studies where it appears that a substantial number of participants are homeless or described as at risk of becoming homeless. As the review is based on an evidence and gap map (EGM) focused on people who are homeless, we should safely assume that the populations included in the studies in the EGM entirely or mainly comprise people who are in fact homeless.

#### 3.1.2 Phenomenon of interest

Given the varying definitions of what constitutes psychosocial interventions, whether interventions are labelled as psychosocial in approach, and the significant operational differences in whether and how these interventions are structured and delivered, it is important to be clear about the types of interventions that we will cover.

The review is focused on formal psychosocial interventions used with adults experiencing homelessness. Interventions based solely or mainly on pharmacological approaches or approaches other than psychosocial, will be excluded. The National Institute for Health and Care Excellence (NICE) provides some help here, stating that formal psychosocial interventions include: contingency management, behavioural couples therapy, community reinforcement approach, social behaviour network therapy, cognitive behavioural relapse prevention‐based therapy, and psychodynamic therapy (NICE, 2007).

We draw on this and have developed a typology of psychosocial interventions to help focus this review. As part of the scoping work to develop this protocol, the typology (Table 1) was discussed and validated with an expert panel of academics, policy makers, experts by experience, and practitioners involved in psychosocial interventions targeted at people experiencing homelessness, held in November 2021. This typology will be developed further during the early stages of this systematic review, as individual studies are categorised against the typology. The primary purpose of this, is to categorise studies for eligibility purposes, and for the effectiveness review, to structure the individual interventions analysis.

**Table 1:** Proposed typology of psychosocial interventions used with adults experiencing homelessness

Category	Low intensity	High intensity
Talking therapy	Brief interventions	Motivational interviewing
	Brief motivational intervention	Motivational enhancement therapy
	Skills training	Cognitive behavioural therapy
		Dialectical behaviour therapy
		Family therapy/couples therapy/community reinforcement
		Therapeutic communities/residential rehabilitation
		Social behaviour and network therapy
		Psychodynamic therapy
		Relapse prevention
		Mentalisation‐Based Therapy
	12‐step facilitation therapy	
Behavioural incentives	Contingency management	
	Cue exposure treatment	
	Non‐contingent rewards	
Self efficacy/		12 step programmes
Self help/mutual aid		SMART

The typology categorises specific interventions as either low intensity or high intensity, drawing on the distinction made by the Welsh Government (2011) between interventions normally delivered as a single session, and interventions that are formal and structured and delivered over a number of sessions.

The typology further categories interventions by their type, as detailed here below in text and in the table that follows.

Talking therapies are a type of psychosocial intervention that primarily involves the service user discussing issues around their thoughts, feelings, or behaviours with a professional therapist. These interventions might be delivered in group or one‐to‐one settings.

Behavioural incentives are a type of psychosocial intervention that use extrinsic rewards or negative consequences to change an individual's behaviour. Finally, self‐help interventions are a group of psychosocial interventions in which individuals work through therapeutic materials or processes on their own, or with minimal input from a professional therapist. This can involve working in a group with others also going through the same process.

#### 3.1.3 Design

Eligible studies will include those that use individual and group interviews, focus groups, observation, or other qualitative‐related methods focused on the experiences, views, or opinions of adults experiencing homelessness.

#### 3.1.4 Evaluation

Types of study will include those where empirical data presenting the experiences, views, or opinions of people who are homeless or at risk of homelessness when accessing or using psychosocial interventions, and are directly presented either as direct quotes or summaries, or as reports of participant experience by researchers.

#### 3.1.5 Research type

Eligible studies will include data reported either as part of a mixed methods study or collected in a qualitative empirical study identified in process evaluations of psychosocial interventions focused on people who are homeless.

### 3.2 Search methods for identification of studies

Studies considered for inclusion in this review will be identified in three ways:

- the Homelessness Implementation Studies Evidence and Gaps Map (White & Narayanan, 2021) (set out below);
- call for evidence (set out in the section ‘searching other sources’ below); and
- hand searches (set out in the section ‘searching other sources’ below).

#### 3.2.1 The homelessness implementation studies EGM

Studies included in this review will be based on an existing Implementation EGM which was last updated in early 2021 by The Campbell Collaboration (White & Narayanan, 2021) and will be updated again before this review is published. The EGM includes 275 qualitative evaluations of interventions aimed at people experiencing, or at risk of experiencing, homelessness in high income countries. (This will be the number of studies from the EGM that will be initially screened for inclusion in this review on title and abstract.) The map is based on a comprehensive three stage search and mapping process. Stage one was to map the included studies in an existing Campbell review on homelessness (Munthe‐Kaas et al., 2018). Stage two was a comprehensive search of 17 academic databases (listed in the Supporting Information: Appendix), three evidence and gap map databases and eight systematic review databases for primary studies and systematic reviews. Stage three included web searches for grey literature, scanning reference lists of included studies, and consultation with experts to identify additional literature. Sample search terms can be found in the protocol (White et al., 2020a). The detailed protocol for the development of the maps is available *here*. A further update to the Implementation EGM will commence in early 2022.

The 2021 edition added 63 studies to the map. It also excluded 34 studies after rescreening, mostly because those studies were impact evaluations which had insufficient implementation evidence to be included. The 2021 edition therefore hosts 275 studies whereas the 2018 edition hosted 246 studies. In the first edition of the implementation map, around 56% of the studies included were from North America and 25% from the UK. The proportion of UK‐based studies in the new version of the implementation map increased by 2% compared to the previous edition (for a total of 73 studies).

The EGM includes systematic reviews and evidence reviews, as well as primary studies. The review team will unpack any systematic review or evidence review, and cross check the unpacked primary studies against the EGM. The review team will undertake a title and abstract review on any unpacked primary study that is not included in the EGM. (And any unpacked primary study, i.e., in the EGM will be included in the title and abstract review of the *n* = 275 EGM studies.)

The review team will work closely with the Campbell Collaboration team undertaking this EGM update to ensure that studies covered in this proposed review are based on up‐to‐date searches.

#### 3.2.2 Grey literature

In addition to the above, to build the EGM, the Campbell team undertook additional website searches for grey literature, including those of bilateral and multilateral organisations. A major source of these studies is the Canadian Homeless Hub. (www.homelesshub.ca/). The Campbell EGM team also searched the websites of government departments (e.g., Housing and Urban Development in the United States) as well as state or county governments in Australia, Canada, the United States, the United Kingdom, and major cities in these countries. In addition, the websites of homelessness agencies, such as Crisis, Homeless Link, Shelter in the United Kingdom and Mission Australia and other relevant databases from around the world as listed in Supporting Information: Appendix A, were searched. For these searches we either navigated to the relevant page listing studies, or searched either the site as a whole or the publications page, using a simple search string of ‘homeless evaluation’. The website results were screened online, with the proposed included studies checked by a second screener.

#### 3.2.3 Searching other resources

In January/February 2022 the review team undertaking the review set out in this protocol issued a *call for grey evidence* (with a deadline of 28th February 2022) which was disseminated through Manchester Metropolitan University and the Centre for Homelessness Impact social media channels, inviting people with lived experience, researchers, commissioners, service providers and wider stakeholders to submit relevant grey literature evidence for consideration in both these parallel reviews. Specifically, the call was for evidence that is:

- empirical, based on research that:
  ○ gives voice to people with experience of homelessness;
  ○ measures the impact of interventions (before and after, quasi‐experimental, randomised controlled trial);
  ○ identifies the experiences of accessing and using, interventions;
- is about psychosocial interventions aimed at preventing or reducing homelessness, mental ill‐health, and problematic substance use;
- is not published in a book or academic journal; and
- is specific to the UK, or England, Northern Ireland, Scotland or Wales.

The reviewers will also *hand search key journals*, using similar search terms and date ranges as White et al. (2020a). While some may have already been searched as part of the evidence and gap map (White et al., 2020a, 2020b; Singh & White, 2022), this targeted journal search and more substance use and treatment focused search will further ensure the capture of all existing literature. The hand searched journals will include:

- Psychiatric Services Journal
- American Journal of Public Health
- BMJ
- European Journal of Homelessness
- Housing Studies
- Social Policy and Administration
- Journal of Social Distress and Homelessness

#### 3.2.4 Title and abstract screening

#At this stage, a list of studies will be available for title and abstract screening. This list will be include:

- 275 studies in the Implementation EGM;
- additional studies^1^ identified through the unpacking of systematic and evidence reviews included in the EGM;
- additional studies identified through hand searches; and
- additional studies identified through the call for evidence.

The studies identified from the four sources listed above will be screened in two stages; (i) title/abstract, (ii) full‐text using the inclusion/exclusion criteria for this review (the inclusion and exclusion criteria are listed in the Supporting Information: Appendix). All screening will be undertaken by two reviewers, and any disagreements will be discussed with another member of the review team. Twenty‐five percent (25%) of final screening decisions will be sampled by a third reviewer. Final decisions about inclusion will be made by all members of the review team.

### 3.3 Data collection and analysis

#### 3.3.1 Data extraction and management

*Descriptive data* will be extracted from eligible studies in two ways. Some relevant descriptive data will have already been extracted by the Campbell team when developing the EGM and we propose to use these where available. For descriptive data that is not included in the EGM, data will be extracted from studies by two reviewers.

Descriptive data will cover the details of the study, description of the theory of change underpinning the intervention, description of the intervention, qualitative data collection method used, qualitative analysis method used, and confidence in the study's findings (using the Campbell Collaboration's critical appraisal tool for primary studies White and Narayanan, 2021, p. 60). Coding disagreements will be discussed and if necessary passed to a third reviewer for resolution. We will extract data for the following:

– Publication details (e.g., authors, year, source, study location)
– Geographical location—this data is available from the EGM
– Setting
– Intervention details, including basis, focus, typology classification, explicit theory of change
– Participant details, including classification (e.g., age, gender, ethnicity, disability, whether service user)
– Number of service users involved in the study
– Research aim and design
– Service/intervention outcomes

Findings data will also be extracted. These findings data will be used in the thematic synthesis analysis to address the research questions set out above. Findings data will be extracted by two reviewers independently. Extracted data will be compared, and any areas of disagreement will be discussed with another member of the team to achieve consensus. Once consensus has been achieved, extract data will be uploaded to nVivo v12 for line‐by‐line coding (first stage of analysis) for thematic synthesis analysis of participant experience data, and subsequently used for Stages two and three of the thematic synthesis analysis.

#### 3.3.2 Quality assessment/risk of bias

Eligible studies that are included in the Implementation EGM have already been assessed using Campbell's Critical Appraisal Tool for Primary Studies (Singh & White, 2022, p. 60). For any additional studies identified through either the handsearch of relevant journals or the call for grey literature, we will conduct an assessment of confidence in the findings using this tool. For these additional studies, classifications will be undertaken using Campbell's Critical Appraisal Tool for Primary Studies by one researcher and judgements (high/medium/low confidence) will be verified by a second researcher who will sample check 25%. (In the event that there is disagreement on more than 1/3rd of the 25% sample check, two reviewers will independently appraise each of the additional included studies, and any areas of disagreement will be adjudicated by a third independent reviewer.) We will report the outcome of the quality assessments included in the EGM and any additional assessments undertaken by the review team.

#### 3.3.3 Confidence in cumulative findings

The Grading of Recommendations, Assessment, Development and Evaluation—Confidence in the Evidence from Reviews of Qualitative Research (GRADE‐CERQual) (Lewin et al., 2018) will be used to assess levels of confidence on the findings of this qualitative evidence synthesis. This involves assessment across four domains: (1) methodological limitations, (2) coherence, (3) adequacy of data, and (4) relevance. This assessment will be undertaken independently by two reviewers, and any areas of disagreement will be discussed with a third member of the review team to achieve a consensus. We will present these findings in a table including a summary of each finding, confidence in that finding, and an explanation for the rating.

#### 3.3.4 Analysis

We will undertake thematic synthesis analysis of qualitative data of the participants' experience that is directly reported by adults experiencing homelessness when accessing or using psychosocial interventions, drawing on qualitative data from mixed methods evaluation studies and from qualitative studies. Where it is not clear whether a quote comes from a person experiencing homelessness or not, we will flag for discussion by the review team. At least two reviewers will review all text flagged and will include or exclude based on consensus.

We propose to use thematic synthesis (Thomas & Harden, 2008). Thematic synthesis is a research synthesis approach that has been used in several systematic reviews on homelessness, including a recent systematic review of the barriers and facilitators experienced by homeless women accessing antenatal services (McGeough et al., 2020), and of the challenges faced by people experiencing homelessness when accessing palliative care (Hudson et al., 2016).

Thomas and Harden (2008) outline three analytic and inductive steps taken in thematic synthesis. The first step involves line‐by‐line coding of the data of the findings of primary studies. The second step involves organising coded material into related areas to construct themes. These themes will describe the experiences of homeless adults as they engage in psychosocial interventions. The third step in thematic synthesis is ‘the most difficult to describe and, potentially, the most controversial’ (Thomas & Harden, 2008). It involves iteratively examining the descriptive themes, drawing out and inferring from these themes the experiences of homeless adults, and the implications arising from these. Where data point to any aspect that might impact the theory of change, this will be incorporated into the analysis.

Selected studies will be uploaded to nVivo v12 for data extraction and thematic analysis. For data extraction and Stages 1 and 2 of the analysis, two researchers will independently code selected studies. The reviewers will then compare the coded material (what has been coded, and what codes have been used) to identified any areas of disagreement. In any case where the coders disagree, a third researcher will then review the outcome of this discussion to develop a consensus. At the third stage of analysis, two researchers will independently examine and then discuss the key descriptive themes. These will then be drawn out and translated, to infer analytical and more abstract themes. At this stage, the analytical themes will be explored through the lens of the theory of change developed during the design stage of the two reviews, recognising the distinction between ‘data’ driven descriptive themes, and ‘theory’ driven analytic themes (Thomas & Harden, 2008). This will enable us to use the thematic review to generate hypotheses that can be tested against the findings of the effectiveness review, as part of the third stage of analysis (Thomas & Harden, 2008).

#### 3.3.5 Experiences

The core focus of this review is to understand the experiences of, views about, and opinion of adults experiencing homelessness when they (a) access and (b) use psychosocial interventions. The current evidence clearly demonstrates that adults experiencing homelessness have significantly higher levels of mental ill‐health, problematic substance use, and housing instability compared to the general population, and are also less likely to be able to access services (particularly general healthcare) to reduce the impact of, and negative effects from, these experiences. By focusing this review on the experiences of adults experiencing homelessness, the review aims to elevate the voice of? homeless people and thereby address a fundamental limitation of many systematic reviews—that of treating service users as passive research participants rather than as citizens who may actively participate in their own recovery. As part of the broader evidence synthesis (of which this proposed review is a one part, alongside an effectiveness review), the review team will use extracted data on theories change underpinning the interventions covered by the included studies to help understand the causal mechanisms and pathways by which psychosocial interventions are intended or expected to lead to positive change in the core outcomes for adults experiencing homelessness. The review team hopes that these theories will enable synthesis of the effectiveness and experiences reviews, so as to provide in‐depth insight into what works, why, how, for whom, and in what contexts, for psychosocial interventions with this population.

#### 3.3.6 Timeframe for review

The NIHR evidence synthesis research grant covers an 18‐month period from October 2021 to May 2023. To comply with the grant requirements, we plan to submit the final review by no later than May 2023.

#### 3.3.7 Plans for updating the review

Dependent on additional funding.

## CONTRIBUTIONS OF AUTHORS

Lead: Chris O'Leary

Content: Chris O'Leary and Esther Coren

Systematic review methods: Esther Coren

Information retrieval: Anton Roberts and Chris O'Leary

## CONFLICT OF INTEREST

The authors declare that there are no conflicts of interest.

## Supporting information

Supporting information.

## Footnotes

1 That is, studies that are not already included in the EGM
